# A Case of Hemophagocytic Syndrome due to Tuberculosis: Uncommon Manifestation of a Common Disease

**DOI:** 10.1155/2014/613845

**Published:** 2014-10-27

**Authors:** Arijit Singha, Adreesh Mukherjee, Riddhi Dasgupta, Tapas Das

**Affiliations:** Department of Medicine, Institute of Post Graduate Medical Education and Research and SSKM Hospital, 242 AJC Bose Road, Kolkata, West Bengal 700020, India

## Abstract

Hemophagocytic syndrome, also known as hemophagocytic lymphohistiocytosis (HLH), is the manifestation of immune dysregulation. It is associated with ineffective but exaggerated immune response and infiltration of active lymphocytes and histiocytes in various organs. This devastating clinical condition has myriad of clinical and biochemical manifestations such as fever, splenomegaly, pancytopenia, hypertrygliceridemia, and hyperferritinemia. It can be either primary or secondary. Primary HLH usually presents in childhood. Secondary HLH occurs due to infection mostly viruses but other aetiologies are also important as early detection and treatment may improve survival. Hemophagocytosis due to tuberculosis is uncommon. Only handful of cases have been reported mostly in immunocompromised patients. We report a case of hemophagocytic syndrome associated with disseminated tuberculosis in an immunocompetent women highlighting early diagnosis and treatment is a demanding need in this devastating disease.

## 1. Introduction

Hemophagocytic syndrome properly known as hemophagocytic lymphohistiocytosis (HLH) is a clinical condition characterized by ineffective but exaggerated immune response with infiltration of activated histiocytes and lymphocytes in various organs [1]. This life threatening syndrome is associated with myriad of clinical and biochemical features of which fever, splenomegaly, cytopenia, hypertriglyceridemia, and hyperferritinemia are important. Generally HLH is classified into primary (genetic) or secondary (infection, autoimmune, or malignancy). Although viral infection is most commonly associated with hemophagocytic syndrome other etiologies are also important because early treatment may lead to improved survival [2]. We report a case of hemophagocytic syndrome associated with disseminated tuberculosis in an immunocompetent women highlighting early diagnosis and treatment is a demanding need in this devastating disease.

## 2. Case Presentation 

A 35 yr old housewife, not known to be diabetic and hypertensive, was admitted in our hospital with history of intermittent low to moderate grade fever without any chills and rigor and yellowish discoloration of both eyes for last 1month and generalized swelling of abdomen for same duration. Patient had history of intermittent cough with sputum production in the past. There was no history of respiratory distress, abdominal pain, haemoptysis, joint pain, photophobia, any rash, joint swelling, chest pain, palpitation, paroxysmal nocturnal dyspnea, and facial swelling. There was no past history of jaundice, tuberculosis, or any contact, any significant family history, or drug intake. On examination patient was conscious, alert, and oriented. Her pulse rate was 102/min, BP-110/70 mm of Hg, with mild pallor, moderate icterus, and pedal edema bilaterally without evidence of clubbing or jugular venous engorgement.

Systemic examination showed palpable hepatomegaly 2 cm below right subcostal margin and splenomegaly 1.5 cm below left sub costal margin. Clinically ascites was found. There was no lymphadenopathy. During the stay in hospital patient progressively deteriorated with drowsiness and pedal swelling, ascites increased in 2-3 days. There was absence of seizure, respiratory distress, and melena. Patient became drowsy but was oriented to time, place, and person. CNS examination did not show any abnormality (Figure 2).

Subsequently laboratory investigations showed a total bilirubin of 26 mg/dL (conjugated—15.7 mg/dL), total protein 4.1 mg/dL (albumin—2.1 mg/dL), ALP—83 U/L (35–104), SGOT—650 U/L, SGPT—330 U/L, TLC—2800, N76%, Hb—8.2%, and platelet—62000/mm^3^ (pancytopenia). Sputum for AFB was negative while cultures for mycobacterium were sent and the reports were awaited. The workup for malaria, autoimmune conditions like SLE, and rheumatoid arthritis and the direct Coombs test were negative. Regarding viral markers hepatitis A, B, C, and E, HIV1 and HIV2, CMV, and EBV were negative. Prothrombin time was prolonged and serum LDH level was elevated (3042 U/L). Serum triglyceride was also elevated up to 370 mg/dL (30–200 mg/dL). Other biochemical parameters such as serum electrolytes, urea, creatinine, and 24 hr urinary protein were within normal limit. Ascitic fluid study showed a low SAAG ascites with few neutrophils, lymphocytes without any evidence of malignant cells or AFB. Serum ceruloplasmin level was within normal limit (35.68 U/L) and Mantoux test was negative.

**Figure 1:**
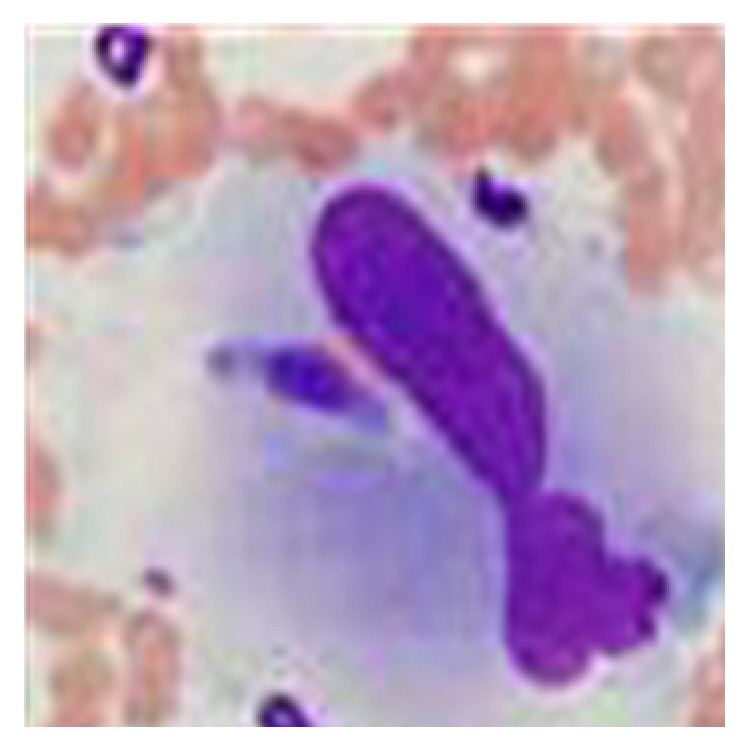
Bone marrow biopsy showing engulfment of erythrocytes by macrophages in oil immersion 40x.

**Figure 2:**
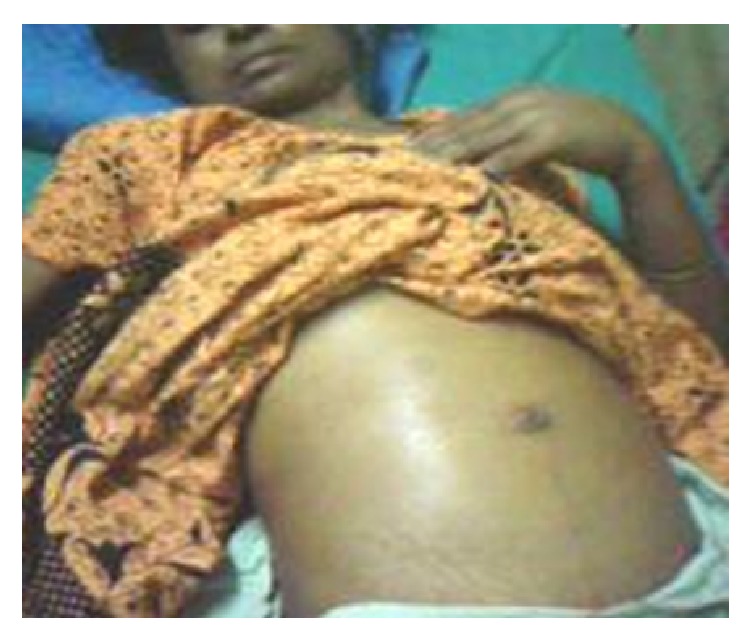
Patient with ascites.

USG of the abdomen showed mild splenomegaly and hepatomegaly with normal echo texture of the liver without any other abnormalities. Chest X-ray was normal. Contrast enhanced CT of abdomen showed only a mildly enlarged liver without any other mass or lymphadenopathy. We did bone marrow aspiration biopsy and found dyserythropoietic cells and engulfment of erythroblast by macrophages (Figure 1). No LD bodies or AFB were found in the bone marrow. However marrow culture was positive for mycobacterium tuberculosis.

As patient had prolonged prothrombin time liver biopsy and splenic biopsy were postponed. At this point the sputum for mycobacterium tuberculosis culture was also reported as positive.

Considering the diagnosis of hemophagocytic syndrome due to disseminated tuberculosis as the most likely possibility, the patient was treated with intravenous methylprednisolone (1 mg/kg/day) for 3 days followed by oral prednisolone in a gradually tapering dose and was initiated on antitubercular drug therapy. Patient showed clinical improvement after 2 days in the form of improved mental status and became afebrile. After 3 days patient had increased appetite, decreased serum bilirubin, serum LDH levels, and decreased ascites (measured by daily weight, abdominal girth). Patient was discharged in stable condition with tapering dose of prednisolone and antitubercular drugs.

## 3. Discussion

Hemophagocytic syndrome is a life-threatening immune dysregulatory syndrome caused by severe hypercytokinemia due to a highly stimulated but ineffective immune process. Despite recent gain in knowledge the pathogenesis of HLH is unclear. However, it is clear that the clinical manifestations of HLH are due to hyperactivation of CD8+ T lymphocytes and macrophages; proliferation, ectopic migration, and infiltration of these cells into various organs; hypercytokinemia with persistently elevated levels of multiple proinflammatory cytokines resulting in progressive organ dysfunction that may lead to death.

These interrelated factors underlie the clinical manifestations of prolonged fever, hepatosplenomegaly, bleeding, skin rash, CNS abnormalities, jaundice, and the laboratory findings of bicytopenia or pancytopenia, coagulopathy, hyperlipidemia, hypofibrinogenemia, hyperferritinemia, transaminitis, hyperbilirubinemia, and hypoalbuminemia [3]. HLH can be primary (familial or genetic) and secondary. Primary HLH usually present in childhood particularly <2 years of age [4], though late presentations usually in 9 to 17 years have been reported. Secondary HLH occurs due to infection mostly viruses. Where primary HLH is associated with perforin mutation, secondary HLH is associated with production of high levels of activating cytokines by host lymphocytes and monocytes. There is no test available that can differentiate between primary and secondary HLH. Course of the disease in both groups is not so varied but secondary HLH has good outcome if detected early [5].

To diagnose HLH 5 out of 8 of the following criteria set up by Histiocyte Society 2004 must be present [6]. Our patient fulfils 5 criteria as she had fever, splenomegaly, hypertriglyceridemia, pancytopenia, and bone marrow evidence of hemophagocytosis.

Hemophagocytosis due to mycobacterium tuberculosis is unusual. To our knowledge only a handful cases have been reported. In their article Brastianos et al. showed most of the patients are immunocompromised either on hemodialysis or HIV infected or renal transplant recipients [7]. Our patient did not have immunocompromised state as per the laboratory data are concerned. What is peculiar in this case is tuberculous infection presented as hemophagocytic syndrome in an immunocompetent patient. HLH due to tuberculosis has high mortality rate (50%). Most of case report reveals death of the patient [8]. In our experience, judicious case detection and timely management have important prognostic significance.

Tuberculosis is very common worldwide and particularly in India. Tuberculosis may present with pancytopenia, features of hemophagocytosis in bone marrow, and negative Mantoux test does not rule out the diagnosis. In spite of high mortality in HLH due to tuberculosis early detection and prompt management may save the patient.
